# 4-[3-(Trifluoro­meth­yl)phen­yl]-5,6,7,8-tetra­hydro­cinnolin-3(2*H*)-one

**DOI:** 10.1107/S1600536808000871

**Published:** 2008-01-18

**Authors:** Xin Wang, Xiao-Mao Zou, You-Quan Zhu, Xu-Hong Hu, Hua-Zheng Yang

**Affiliations:** a State Key Laboratory and Institute of Elemento-Organic Chemistry, Nankai University, Tianjin 300071, People’s Republic of China

## Abstract

The title compound, C_15_H_13_F_3_N_2_O, contains one benzene ring, one cyclo­hexane ring and a pyridazine ring. The dihedral angle formed by the pyridazine ring with the benzene ring is 61.5 (2)°. The crystal structure is stabilized by two inter­molecular hydrogen bonds (N—H⋯O and C—H⋯F). The cyclohexane ring adopts a screw-boat conformation. The CF_3_ group is disordered over two positions; the site occupancy factors are *ca* 0.6 and 0.4.

## Related literature

For related literature, see: Heinisch & Kopelent (1992 ▶); Kolar & Tisler (1999 ▶).
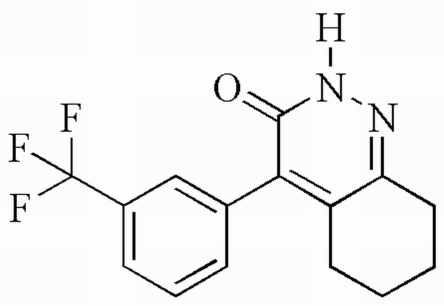


## Experimental

### 

#### Crystal data

C_15_H_13_F_3_N_2_O
*M* *_r_* = 294.27
Monoclinic, 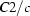

*a* = 8.929 (3) Å
*b* = 11.443 (4) Å
*c* = 27.448 (8) Å
β = 94.232 (6)°
*V* = 2796.6 (15) Å^3^
*Z* = 8
Mo *K*α radiation
μ = 0.12 mm^−1^
*T* = 294 (2) K
0.22 × 0.20 × 0.16 mm

#### Data collection

Bruker SMART CCD area-detector diffractometer
Absorption correction: multi-scan (*SADABS*; Sheldrick, 1996 ▶) *T*_min_ = 0.975, *T*_max_ = 0.982
7053 measured reflections
2485 independent reflections
1098 reflections with *I* > 2σ(*I*)
*R*_int_ = 0.061

#### Refinement

*R*[*F* ^2^ > 2σ(*F* ^2^)] = 0.068
*wR*(*F* ^2^) = 0.211
*S* = 1.02
2485 reflections
223 parameters
85 restraints
H atoms treated by a mixture of independent and constrained refinement
Δρ_max_ = 0.32 e Å^−3^
Δρ_min_ = −0.27 e Å^−3^

### 

Data collection: *SMART* (Bruker, 1999 ▶); cell refinement: *SAINT* (Bruker, 1999 ▶); data reduction: *SAINT*; program(s) used to solve structure: *SHELXS97* (Sheldrick, 2008 ▶); program(s) used to refine structure: *SHELXL97* (Sheldrick, 2008 ▶); molecular graphics: *SHELXTL* (Sheldrick, 2008 ▶); software used to prepare material for publication: *SHELXTL*.

## Supplementary Material

Crystal structure: contains datablocks global, I. DOI: 10.1107/S1600536808000871/at2529sup1.cif

Structure factors: contains datablocks I. DOI: 10.1107/S1600536808000871/at2529Isup2.hkl

Additional supplementary materials:  crystallographic information; 3D view; checkCIF report

## Figures and Tables

**Table 1:** Hydrogen-bond geometry (Å, °)

*D*—H⋯*A*	*D*—H	H⋯*A*	*D*⋯*A*	*D*—H⋯*A*
N2—H2⋯O1^i^	0.91 (4)	1.88 (4)	2.783 (5)	178 (5)
C12—H12⋯F3′^ii^	0.93	2.51	3.362	152

Symmetry codes: (i) 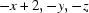; (ii) 
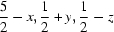
.

**Fig. 1.**
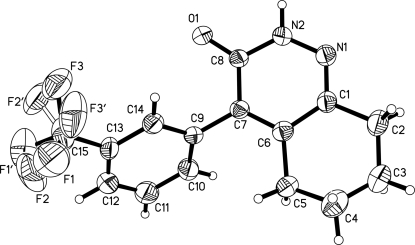
View of the title compound with 30% probability ellipsoid.

**Fig. 2.**
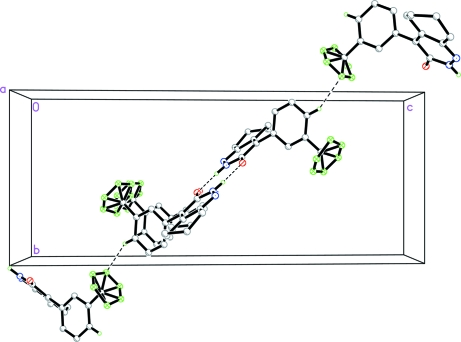
Intermolecular hydrogen-bonding interactions (dashed lines) in the structure of (I). H atoms not involved in hydrogen bonding have been omitted.

## supplementary crystallographic information

### Comment

Many pyridazine derivatives have been found to exhibit biological activities such as insecticidal, fungicidal, herbicidal, plant-growth regulatory activity, *etc*. (Heinisch & Kopelent, 1992). For example, pyridate, credazine and maleic hydrazide (Kolar & Tisler, 1999) have been commercialized as herbicides. In order to discover new biologically active pyridazine compounds, the title compound, (I), was synthesized and its structure is reported here.

In the molecule of (I) (Fig. 1), the central pyridazine ring (C1—C8/N1/N2) is approximately coplanar with the cyclohexane ring (C1—C6) [dihedral angle = 4.36 (29)°] and the largest deviation from the mean plane is 0.306 (6) Å for atom C4. The dihedral angle formed by the heterocycle and the benzene ring (C9—C14) is 61.50 (18)°. The molecule is further stabilized by intermolecular N—H···O and C—H···F hydrogen bonds(Table 1). Glide-related molecules are linked *via* C—H···F hydrogen-bonded chains along the *c* axis. Part of the chain structure is shown in Fig. 2.

### Experimental

4-(3-(Trifluoromethyl)phenyl)-4,4a,5,6,7,8-hexahydrocinnolin-3(2*H*)-one (1.5 mmol), and 0.5 g anhydrous copper(II) chloride were mixed in acetonitrile (40 ml), refluxed for 2 h. Water (20 ml) were then added. The organic layer was washed successively with saturated sodium bicarbonate solution and brine, dried over anhydrous magnesium sulfateThe solvent was then evaporated in vacuo. The residue was purified *via* column chromatography. single crystals of (I) suitable for X-ray analysis were grown from ethyl acetate and petroleum ether at room temperature.

### Refinement

The trifluoromethyl group shows positional disorder. At the final stage of the refinement, the occupancy factors of two possible sites, C15/F1/F2/F3 and C15/F1'/F2'/F3', were fixed at 0.429 and 0.571 respectively. All H atoms were positioned geometrically, with C—H = 0.93 and 0.97 A° and N—H = 0.91 (4) A°, and included in the final cycles of refinement using a riding model, with *U*_iso_(H) = 1.2*U*_eq_(C).

### Crystal data

**Table:**

C_15_H_13_F_3_N_2_O	*F*_000_ = 1216
*M**_r_* = 294.27	*D*_x_ = 1.398 Mg m^−^^3^
Monoclinic, *C*2/*c*	Mo *K*α radiation λ = 0.71073 Å
Hall symbol: -C 2yc	Cell parameters from 1012 reflections
*a* = 8.929 (3) Å	θ = 2.9–20.2º
*b* = 11.443 (4) Å	µ = 0.12 mm^−^^1^
*c* = 27.448 (8) Å	*T* = 294 (2) K
β = 94.232 (6)º	Prism, colourless
*V* = 2796.6 (15) Å^3^	0.22 × 0.20 × 0.16 mm
*Z* = 8	

### Data collection

**Table:**

Bruker SMART CCD area-detector diffractometer	2485 independent reflections
Radiation source: fine-focus sealed tube	1098 reflections with *I* > 2σ(*I*)
Monochromator: graphite	*R*_int_ = 0.061
*T* = 294(2) K	θ_max_ = 25.0º
φ and ω scans	θ_min_ = 1.5º
Absorption correction: multi-scan(SADABS; Sheldrick, 1996)	*h* = −10→10
*T*_min_ = 0.975, *T*_max_ = 0.982	*k* = −13→8
7053 measured reflections	*l* = −32→32

### Refinement

**Table:**

Refinement on *F*^2^	Secondary atom site location: difference Fourier map
Least-squares matrix: full	Hydrogen site location: inferred from neighbouring sites
*R*[*F*^2^ > 2σ(*F*^2^)] = 0.068	H atoms treated by a mixture of independent and constrained refinement
*wR*(*F*^2^) = 0.212	*w* = 1/[σ^2^(*F*_o_^2^) + (0.0738*P*)^2^ + 4.8514*P*] where *P* = (*F*_o_^2^ + 2*F*_c_^2^)/3
*S* = 1.02	(Δ/σ)_max_ = 0.001
2485 reflections	Δρ_max_ = 0.32 e Å^−^^3^
223 parameters	Δρ_min_ = −0.27 e Å^−^^3^
85 restraints	Extinction correction: SHELXL97 (Sheldrick, 1997), Fc^*^=kFc[1+0.001xFc^2^λ^3^/sin(2θ)]^-1/4^
Primary atom site location: structure-invariant direct methods	Extinction coefficient: 0.0029 (8)

### Special details

**Table:**

Geometry. All e.s.d.'s (except the e.s.d. in the dihedral angle between two l.s. planes) are estimated using the full covariance matrix. The cell e.s.d.'s are taken into account individually in the estimation of e.s.d.'s in distances, angles and torsion angles; correlations between e.s.d.'s in cell parameters are only used when they are defined by crystal symmetry. An approximate (isotropic) treatment of cell e.s.d.'s is used for estimating e.s.d.'s involving l.s. planes.
Refinement. Refinement of *F*^2^ against ALL reflections. The weighted *R*-factor *wR* and goodness of fit *S* are based on *F*^2^, conventional *R*-factors *R* are based on *F*, with *F* set to zero for negative *F*^2^. The threshold expression of *F*^2^ > σ(*F*^2^) is used only for calculating *R*-factors(gt) *etc*. and is not relevant to the choice of reflections for refinement. *R*-factors based on *F*^2^ are statistically about twice as large as those based on *F*, and *R*- factors based on ALL data will be even larger.

### Fractional atomic coordinates and isotropic or equivalent isotropic displacement parameters (Å^2^)

**Table:**

	*x*	*y*	*z*	*U*_iso_*/*U*_eq_	Occ. (<1)
F1	1.1701 (18)	0.1095 (16)	0.2649 (5)	0.144 (5)	0.429 (9)
F2	1.3627 (15)	0.1988 (12)	0.2422 (6)	0.167 (5)	0.429 (9)
F3	1.274 (2)	0.0347 (12)	0.2066 (5)	0.139 (5)	0.429 (9)
F1'	1.2515 (15)	0.1731 (11)	0.2699 (3)	0.152 (4)	0.571 (9)
F2'	1.3577 (10)	0.0982 (12)	0.2113 (4)	0.130 (4)	0.571 (9)
F3'	1.1498 (13)	0.0330 (10)	0.2327 (5)	0.170 (4)	0.571 (9)
O1	1.0587 (4)	0.0810 (3)	0.05160 (12)	0.0661 (10)	
N1	0.6764 (5)	0.0930 (3)	0.00974 (15)	0.0601 (11)	
N2	0.8244 (5)	0.0709 (4)	0.01511 (15)	0.0579 (11)	
C1	0.6214 (5)	0.1615 (4)	0.04183 (19)	0.0547 (13)	
C2	0.4543 (5)	0.1771 (5)	0.0370 (2)	0.0755 (16)	
H2A	0.4236	0.1960	0.0033	0.091*	
H2B	0.4074	0.1035	0.0446	0.091*	
C3	0.3977 (7)	0.2691 (7)	0.0689 (3)	0.120 (3)	
H3A	0.4001	0.3431	0.0518	0.144*	
H3B	0.2935	0.2522	0.0739	0.144*	
C4	0.4780 (7)	0.2826 (7)	0.1160 (3)	0.103 (2)	
H4A	0.4599	0.2141	0.1356	0.124*	
H4B	0.4373	0.3496	0.1322	0.124*	
C5	0.6424 (6)	0.2986 (5)	0.11490 (19)	0.0708 (16)	
H5A	0.6891	0.2872	0.1476	0.085*	
H5B	0.6622	0.3784	0.1053	0.085*	
C6	0.7142 (5)	0.2159 (4)	0.08030 (17)	0.0533 (13)	
C7	0.8642 (5)	0.1920 (4)	0.08449 (15)	0.0480 (12)	
C8	0.9248 (6)	0.1122 (4)	0.05062 (16)	0.0497 (12)	
C9	0.9705 (5)	0.2428 (4)	0.12244 (17)	0.0539 (13)	
C10	0.9951 (6)	0.3607 (5)	0.1261 (2)	0.0813 (18)	
H10	0.9450	0.4107	0.1036	0.098*	
C11	1.0930 (7)	0.4069 (6)	0.1624 (3)	0.111 (3)	
H11	1.1075	0.4872	0.1647	0.133*	
C12	1.1682 (7)	0.3337 (8)	0.1950 (3)	0.110 (3)	
H12	1.2339	0.3643	0.2196	0.133*	
C13	1.1474 (6)	0.2162 (7)	0.1915 (2)	0.083 (2)	
C14	1.0497 (5)	0.1703 (5)	0.15529 (17)	0.0650 (15)	
H14	1.0369	0.0898	0.1530	0.078*	
C15	1.2323 (9)	0.1368 (8)	0.2247 (2)	0.114 (3)	
H2	0.862 (5)	0.023 (4)	−0.0072 (14)	0.081 (18)*	

### Atomic displacement parameters (Å^2^)

**Table:**

	*U*^11^	*U*^22^	*U*^33^	*U*^12^	*U*^13^	*U*^23^
F1	0.165 (9)	0.167 (9)	0.102 (7)	0.031 (7)	0.032 (7)	0.026 (7)
F2	0.139 (8)	0.198 (9)	0.157 (8)	0.018 (7)	−0.044 (7)	0.025 (7)
F3	0.150 (9)	0.146 (8)	0.118 (7)	0.075 (7)	−0.004 (7)	0.012 (6)
F1'	0.179 (8)	0.207 (8)	0.064 (4)	−0.003 (7)	−0.021 (5)	0.006 (5)
F2'	0.102 (5)	0.166 (8)	0.123 (6)	0.071 (5)	0.011 (5)	0.020 (6)
F3'	0.158 (7)	0.185 (7)	0.157 (7)	0.032 (6)	−0.052 (6)	0.061 (6)
O1	0.057 (2)	0.068 (2)	0.071 (2)	0.0144 (17)	−0.0021 (17)	−0.0177 (19)
N1	0.059 (3)	0.052 (3)	0.068 (3)	0.002 (2)	−0.004 (2)	−0.001 (2)
N2	0.058 (3)	0.053 (3)	0.061 (3)	0.007 (2)	−0.005 (2)	−0.010 (2)
C1	0.053 (3)	0.045 (3)	0.065 (3)	0.000 (2)	−0.004 (3)	0.004 (3)
C2	0.054 (3)	0.071 (4)	0.099 (4)	0.002 (3)	−0.004 (3)	0.003 (4)
C3	0.059 (4)	0.150 (7)	0.147 (7)	0.020 (4)	−0.005 (4)	−0.044 (6)
C4	0.068 (4)	0.135 (6)	0.109 (5)	0.009 (4)	0.021 (4)	−0.014 (5)
C5	0.065 (3)	0.078 (4)	0.070 (3)	0.018 (3)	0.002 (3)	−0.009 (3)
C6	0.059 (3)	0.046 (3)	0.055 (3)	0.006 (2)	0.004 (2)	0.007 (2)
C7	0.056 (3)	0.041 (3)	0.047 (3)	0.006 (2)	0.001 (2)	0.002 (2)
C8	0.055 (3)	0.042 (3)	0.051 (3)	0.005 (2)	0.000 (3)	−0.002 (2)
C9	0.056 (3)	0.056 (3)	0.049 (3)	0.013 (2)	0.000 (2)	−0.009 (3)
C10	0.076 (4)	0.062 (4)	0.102 (5)	0.008 (3)	−0.020 (3)	−0.019 (3)
C11	0.091 (5)	0.085 (5)	0.152 (7)	0.013 (4)	−0.026 (5)	−0.055 (5)
C12	0.074 (4)	0.150 (7)	0.104 (5)	0.032 (5)	−0.020 (4)	−0.063 (6)
C13	0.064 (4)	0.131 (6)	0.051 (3)	0.039 (4)	−0.009 (3)	−0.017 (4)
C14	0.069 (3)	0.076 (4)	0.049 (3)	0.021 (3)	0.002 (3)	−0.002 (3)
C15	0.102 (6)	0.155 (7)	0.080 (5)	0.016 (5)	−0.025 (4)	−0.003 (5)

### Geometric parameters (Å, °)

**Table:**

F1—C15	1.310 (9)	C4—H4A	0.9700
F2—C15	1.416 (9)	C4—H4B	0.9700
F3—C15	1.333 (9)	C5—C6	1.516 (7)
F1'—C15	1.308 (8)	C5—H5A	0.9700
F2'—C15	1.282 (8)	C5—H5B	0.9700
F3'—C15	1.423 (9)	C6—C7	1.363 (6)
O1—C8	1.246 (5)	C7—C8	1.437 (6)
N1—C1	1.302 (6)	C7—C9	1.476 (6)
N1—N2	1.343 (5)	C9—C10	1.370 (7)
N2—C8	1.360 (6)	C9—C14	1.381 (6)
N2—H2	0.91 (4)	C10—C11	1.382 (8)
C1—C6	1.435 (6)	C10—H10	0.9300
C1—C2	1.499 (6)	C11—C12	1.364 (9)
C2—C3	1.481 (8)	C11—H11	0.9300
C2—H2A	0.9700	C12—C13	1.360 (9)
C2—H2B	0.9700	C12—H12	0.9300
C3—C4	1.441 (8)	C13—C14	1.377 (7)
C3—H3A	0.9700	C13—C15	1.459 (9)
C3—H3B	0.9700	C14—H14	0.9300
C4—C5	1.482 (7)		
			
C1—N1—N2	117.2 (4)	C10—C9—C14	118.2 (5)
N1—N2—C8	127.2 (4)	C10—C9—C7	122.0 (5)
N1—N2—H2	117 (3)	C14—C9—C7	119.7 (5)
C8—N2—H2	116 (3)	C9—C10—C11	121.2 (6)
N1—C1—C6	122.2 (4)	C9—C10—H10	119.4
N1—C1—C2	115.8 (5)	C11—C10—H10	119.4
C6—C1—C2	122.0 (5)	C12—C11—C10	119.5 (7)
C3—C2—C1	114.4 (5)	C12—C11—H11	120.2
C3—C2—H2A	108.7	C10—C11—H11	120.2
C1—C2—H2A	108.7	C13—C12—C11	120.2 (6)
C3—C2—H2B	108.7	C13—C12—H12	119.9
C1—C2—H2B	108.7	C11—C12—H12	119.9
H2A—C2—H2B	107.6	C12—C13—C14	120.2 (6)
C4—C3—C2	115.9 (6)	C12—C13—C15	120.6 (7)
C4—C3—H3A	108.3	C14—C13—C15	119.1 (7)
C2—C3—H3A	108.3	C13—C14—C9	120.6 (6)
C4—C3—H3B	108.3	C13—C14—H14	119.7
C2—C3—H3B	108.3	C9—C14—H14	119.7
H3A—C3—H3B	107.4	F2'—C15—F1'	109.0 (9)
C3—C4—C5	115.0 (6)	F2'—C15—F1	126.4 (9)
C3—C4—H4A	108.5	F1'—C15—F1	46.3 (8)
C5—C4—H4A	108.5	F2'—C15—F3	47.0 (8)
C3—C4—H4B	108.5	F1'—C15—F3	127.7 (10)
C5—C4—H4B	108.5	F1—C15—F3	104.7 (12)
H4A—C4—H4B	107.5	F2'—C15—F2	63.7 (8)
C4—C5—C6	113.7 (5)	F1'—C15—F2	58.2 (8)
C4—C5—H5A	108.8	F1—C15—F2	102.9 (11)
C6—C5—H5A	108.8	F3—C15—F2	108.7 (12)
C4—C5—H5B	108.8	F2'—C15—F3'	103.3 (10)
C6—C5—H5B	108.8	F1'—C15—F3'	98.7 (10)
H5A—C5—H5B	107.7	F1—C15—F3'	54.2 (8)
C7—C6—C1	119.0 (4)	F3—C15—F3'	59.2 (9)
C7—C6—C5	122.0 (4)	F2—C15—F3'	142.0 (8)
C1—C6—C5	118.9 (4)	F2'—C15—C13	117.3 (7)
C6—C7—C8	119.2 (4)	F1'—C15—C13	114.9 (8)
C6—C7—C9	123.9 (4)	F1—C15—C13	116.2 (9)
C8—C7—C9	116.9 (4)	F3—C15—C13	117.3 (8)
O1—C8—N2	119.7 (4)	F2—C15—C13	106.0 (8)
O1—C8—C7	125.1 (4)	F3'—C15—C13	111.4 (7)
N2—C8—C7	115.2 (4)		
			
C1—N1—N2—C8	0.0 (7)	C8—C7—C9—C10	119.0 (5)
N2—N1—C1—C6	2.7 (6)	C6—C7—C9—C14	119.2 (5)
N2—N1—C1—C2	−175.5 (4)	C8—C7—C9—C14	−60.3 (6)
N1—C1—C2—C3	−170.9 (5)	C14—C9—C10—C11	−1.9 (9)
C6—C1—C2—C3	10.9 (8)	C7—C9—C10—C11	178.7 (5)
C1—C2—C3—C4	−34.6 (9)	C9—C10—C11—C12	0.9 (10)
C2—C3—C4—C5	52.9 (10)	C10—C11—C12—C13	0.3 (11)
C3—C4—C5—C6	−44.3 (8)	C11—C12—C13—C14	−0.4 (10)
N1—C1—C6—C7	−2.3 (7)	C11—C12—C13—C15	177.2 (7)
C2—C1—C6—C7	175.7 (4)	C12—C13—C14—C9	−0.6 (8)
N1—C1—C6—C5	177.4 (4)	C15—C13—C14—C9	−178.3 (5)
C2—C1—C6—C5	−4.5 (7)	C10—C9—C14—C13	1.8 (8)
C4—C5—C6—C7	−159.9 (5)	C7—C9—C14—C13	−178.9 (5)
C4—C5—C6—C1	20.4 (7)	C12—C13—C15—F2'	−90.7 (11)
C1—C6—C7—C8	−0.6 (6)	C14—C13—C15—F2'	86.9 (12)
C5—C6—C7—C8	179.6 (4)	C12—C13—C15—F1'	39.4 (13)
C1—C6—C7—C9	179.9 (4)	C14—C13—C15—F1'	−142.9 (10)
C5—C6—C7—C9	0.2 (7)	C12—C13—C15—F1	91.1 (14)
N1—N2—C8—O1	177.9 (4)	C14—C13—C15—F1	−91.3 (14)
N1—N2—C8—C7	−2.7 (7)	C12—C13—C15—F3	−144.0 (13)
C6—C7—C8—O1	−177.8 (4)	C14—C13—C15—F3	33.7 (15)
C9—C7—C8—O1	1.7 (7)	C12—C13—C15—F2	−22.5 (12)
C6—C7—C8—N2	2.9 (6)	C14—C13—C15—F2	155.2 (10)
C9—C7—C8—N2	−177.6 (4)	C12—C13—C15—F3'	150.6 (10)
C6—C7—C9—C10	−61.5 (7)	C14—C13—C15—F3'	−31.8 (12)

### Hydrogen-bond geometry (Å, °)

**Table:**

*D*—H···*A*	*D*—H	H···*A*	*D*···*A*	*D*—H···*A*
N2—H2···O1^i^	0.91 (4)	1.88 (4)	2.783 (5)	178 (5)
C12—H12···F3'^ii^	0.93	2.51	3.362	152

Symmetry codes: (i) −*x*+2, −*y*, −*z*; (ii) −*x*+5/2, *y*+1/2, −*z*+1/2.

## References

[bb1] Bruker (1999). *SMART* (Version 5.618) and *SAINT* (Version 6.45). Bruker AXS Inc., Madison, Wisconsin, USA.

[bb2] Heinisch, G. & Kopelent, H. (1992). *Prog. Med. Chem.* **29**, 141–183. 10.1016/s0079-6468(08)70007-9 1475369

[bb3] Kolar, P. & Tisler, M. (1999). *Adv. Heterocycl. Chem.* **75**, 167–241.

[bb4] Sheldrick, G. M. (1996). *SADABS* University of Göttingen, Germany.

[bb5] Sheldrick, G. M. (2008). *Acta Cryst.* A**64**, 112–122. 10.1107/S0108767307043930 18156677

